# Cigarette smoke extract-mediated FABP4 upregulation suppresses viability and induces apoptosis, inflammation and oxidative stress of bronchial epithelial cells by activating p38 MAPK/MK2 signaling pathway

**DOI:** 10.1186/s12950-022-00304-z

**Published:** 2022-06-15

**Authors:** Wei Zhang, Yibin Zhang, Qi Zhu

**Affiliations:** 1 Center for Rehabilitation Medicine, Rehabilitation & Sports Medicine Research Institute of Zhejiang Province, Department of Rehabilitation Medicine, Zhejiang Provincial People’s Hospital (Affiliated People’s Hospital, Hangzhou Medical College), Hangzhou, 310014 Zhejiang China; 2 Department of Respiratory, The Third People’s Hospital of Yuhang District, Hangzhou, 311115 Zhejiang China; 3 Emergency and Critical Care Center, Department of Pulmonary and Critical Care Medicine, Zhejiang Provincial People’s Hospital (Affiliated People’s Hospital, Hangzhou Medical College), No. 158 Shangtang Road, Gongshu District, Hangzhou 310014 Zhejiang, China

**Keywords:** Cigarette smoke extract, Bronchial inflammation, Fatty acid binding protein 4, p38 MAPK/MK2 signaling pathway, Inflammatory response, Cell apoptosis

## Abstract

**Background:**

Long-term inhalation of cigarette smoke is considered to be one of the main causes of bronchial epithelioid cell damage, but its underlying mechanism has to be further clarified.

**Methods:**

Gene expression at mRNA level and protein levels were detected by qRT-PCR and western blot analysis respectively. CCK-8, TUNEL assays, ELISA, western blot analysis and commercial kits were utilized to test cell viability, apoptosis inflammatory response and oxidative stress. The correlation between fatty acid binding protein 4 (FABP4) and the p38 mitogen-activated protein kinase (MAPK)/MAPK activated kinase 2 (MK2) signaling pathway was verified by western blot analysis and rescue assays.

**Results:**

Cigarette smoke extract (CSE) exposure decreased viability, induced apoptosis and inflammatory response in 16HBE cells. Moreover, the expression of FABP4 in CSE-treated 16HBE cells was up-regulated in a time and dose-dependent manner. Ablation of FABP4 in 16HBE cells significantly protected against CSE-mediated cell viability decline and apoptosis. Further, FABP4 knockdown suppressed inflammatory response by down-regulating the elevated levels of cellular inflammatory factors including TNF-α, IL-1β, IL-6, Cyclooxygenase-2 (Cox-2) and inducible nitric oxide synthase (iNOS) in CSE-treated 16HBE cells. The oxidative stress induced by CSE in 16HBE cells was also inhibited by FABP4 silence as evidence by reduced ROS and MDA level but increased SOD activity caused by FABP4 silence. Finally, all the above effects of FABP4 silence on CSE-treated 16HBE cells were reversed by asiatic acid, an agonist of p38 mitogen-activated protein kinase (MAPK).

**Conclusions:**

The up-regulation of FABP4 expression mediated by CSE exerted pro-inflammatory, pro-oxidative stress and pro-apoptotic effects on bronchial epithelial cells by activating the p38 MAPK/MK2 signaling pathway. Our findings help to further understand the underlying mechanism of cigarette smoke-induced bronchial inflammation.

## Background

Tobacco leads to nearly 6 million deaths worldwide each year and is the most preventable cause of morbidity and death [1]. Metallic substances in cigarettes including arsenic, cadmium and lead are transferred through cigarette smoke (CS), accumulated in the lung tissues of smokers and greatly threaten human health [2]. In addition, the potential carcinogenicity of the additives contained in cigarettes cannot be ignored [3]. More importantly, cigarette-smoke exposure (CSE) is one of the common risk factors of chronic obstructive pulmonary disease (COPD), a leading cause of death nationally and worldwide [3]. Besides, CSE exposure contributes to airway remodeling and tumor-like transformation of bronchial epithelial cells [4, 5]. Hence, identifying effective biomarkers in CSE-treated bronchial epithelial cells is of great significance in the therapy for COPD.

Fatty acid binding protein 4 (FABP4) is mainly expressed by pulmonary macrophages, and differentially expressed in endothelial cells of the bronchial microvascular system. It is an intracellular lipid chaperone and adipose factor that regulates metabolic and inflammatory pathways [6–8]. Accumulating studies have exhibited the important regulatory role of FABP4 in human diseases. For example, FABP4 is highly expressed in hyperoxic lung injury and may be implicated in bronchopulmonary dysplasia [9]. FABP4 has been detected to be overexpressed during sepsis-induced acute lung injury, which causes the production of inflammatory cytokines by increasing the level of reactive oxygen species (ROS) [10]. Research by Xiao Na Ge et al. have showed that FABP4 promotes the adhesion and migration of eosinophils and promotes airway inflammation under inflammatory conditions in allergic asthma [11]. Moreover, FABP4 may also be involved in the pathogenesis of COPD. For instance, it is found that FABP4 expression is up-regulated with the increase of GOLD grade in the serum of COPD patients which is related to the up-regulation of serum interleukin 6 (IL-6) levels and the severity of hypoxia [12]. Zhang et al. have proposed that plasma FABP4 levels in females with COPD were significantly increased compared with both males with COPD and healthy females [13]. FABP4 levels also positively correlated with adiponectin and tumor necrosis factor α in COPD patients [13]. On the contrary, another study has revealed that airway FABP4 levels are reduced in COPD patients [14]. Although the above studies reported the controversial effect of FABP4 on COPD, they all suggested the important role of FABP4 in COPD pathogenesis. Hence, we hypothesize that FABP4 may also play a role in CSE-induced bronchial epithelial cell damage.

CSE induces phosphorylation of p38 mitogen-activated protein kinase (MAPK) in human bronchial epithelial cells by phosphorylation of a known downstream substrate of MAPK, MAPK activated kinase 2 (MK2) [15]. Eupatiline, a pharmacologically active flavone, down-regulates levels of phosphorylated P38, extracellular regulated protein kinases (ERK), and c-Jun N-terminal kinase (JNK) for anti-inflammatory activity [16]. What’s more, FABP4 inhibitor BMS309403 is discovered to inhibit p38 MAPK activation and alleviate fatty acid-induced endoplasmic reticulum stress-related inflammation in skeletal muscle [17]. Therefore, we further explore the relationship between FABP4 and the activation of p38 MAPK/MK2 signaling pathway in CSE-mediated bronchial epithelial cells.

Here, the study illustrates that CSE-induced bronchial epithelial cell damage is significantly correlated with FABP4 expression. The up-regulated FABP4 induces inflammatory response in bronchial epithelial cells by activating the p38 MAPK/MK2 signaling pathway, reducing cell viability and even causing cell apoptosis.

## Results

### CSE upregulates FABP4 expression in 16HBE cells

**Fig. 1:**
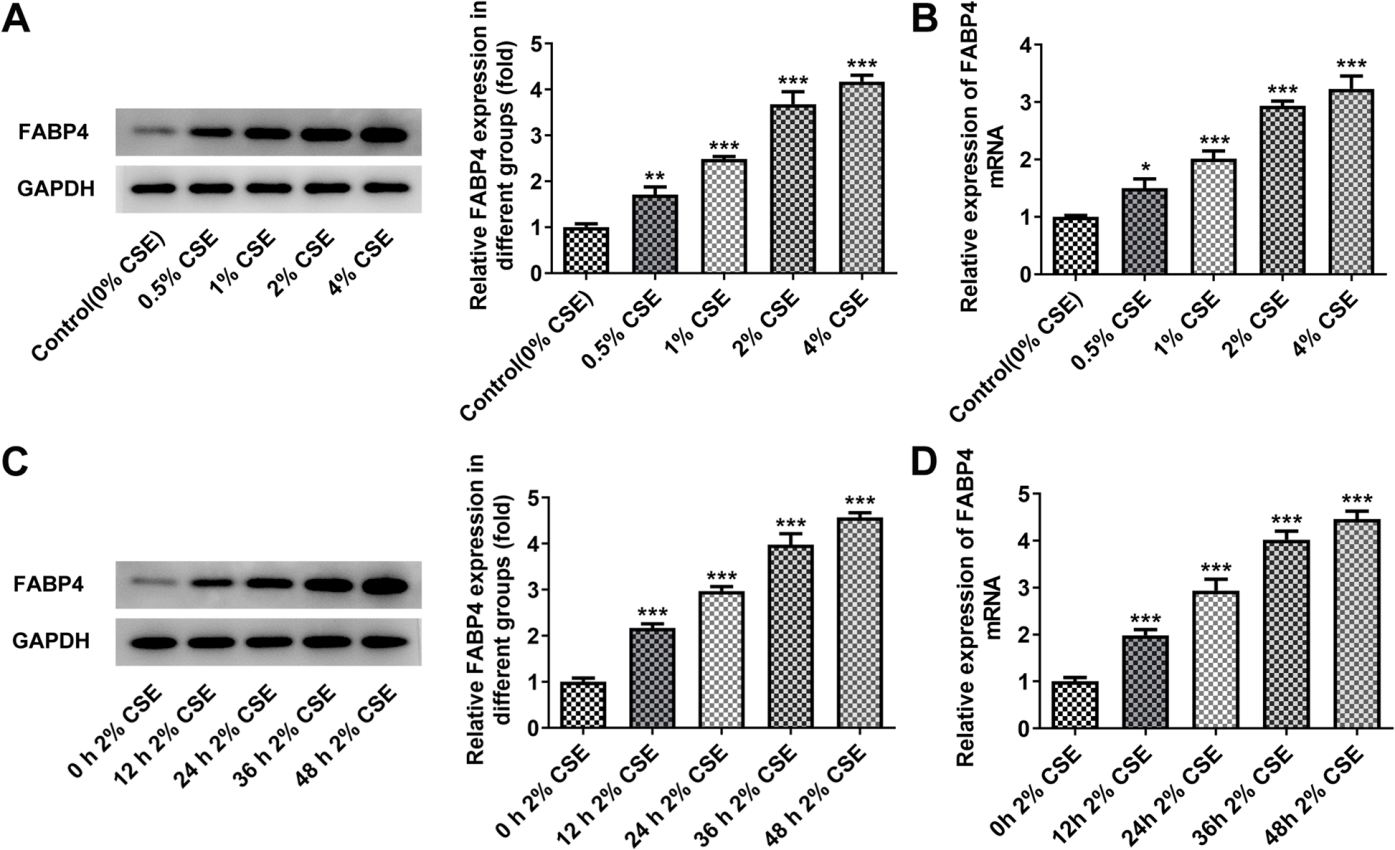
CS mediates the up-regulation of FABP4 in 16HBE cells. **A** 16HBE cells were treated with different doses of CSE (0.5%, 1%, 2% and 4%) for 24 h, and the normal 16HBE cells without CSE treatment were used as control. Representative Western blot images and quantitative analysis, using the rabbit anti-FABP4, showed an incremental increase in the FABP4 expression with CSE. GAPDH served as the loading control. **B** qRT-PCR analysis, using the primer of human FABP4, showed the experimental results consistent with (**A**). **p < 0.01, ***p < 0.001 vs. control group. **C** 16HBE cells were treated with 2% CSE for different time (0 h, 12 h, 24 h, 36 h and 48 h). 2% CSE treatment increased the FABP4 expression in a time dependent manner with a maximum expression at 48 h after the 2% CSE treatment. GAPDH served as the loading control. **D** qRT-PCR analysis, using the primer of human FABP4, showed the experimental results consistent with (**C**). ***p < 0.001 vs. 0 h 2% CSE group. 16HBE, Human bronchial epithelioid cell line; FABP4, Fatty Acid Binding Protein 4; GAPDH, Glyceraldehyde-3-Phosphate Dehydrogenase; CSE, cigarette smoke extract

The 16HBE cells treated with cigarette smoke extract (CSE) showed a progressive upregulation in the FABP4 expression at mRNA level (~ threefold enhancement with the maximum dose) and protein level (~ four fold enhancement with the maximum dose) in a dose-dependent manner from 0.5% CSE to 4% CSE, as compared with the normal 16HBE cells without CSE treatment (Fig. 1A, B). Considering that 2% CSE caused significant increase in FABP4 expression and was widely used for inducing bronchial epithelial cells injury [18, 19], it was subsequently chosen for treat 16HBE cells. Moreover, we noticed the increment of FABP4 expression in a time-dependent manner (0 h, 12 h, 24 h, 36 h and 48 h; Fig. 1C, D) caused by 2% CSE. For further study, we utilized 2% CSE to treat 16HBE cells for 48 h to perform the subsequent experiments.

### shRNA-mediated FABP4 abrogation alleviates CSE-mediated 16HBE cell viability decline and apoptosis

**Fig. 2:**
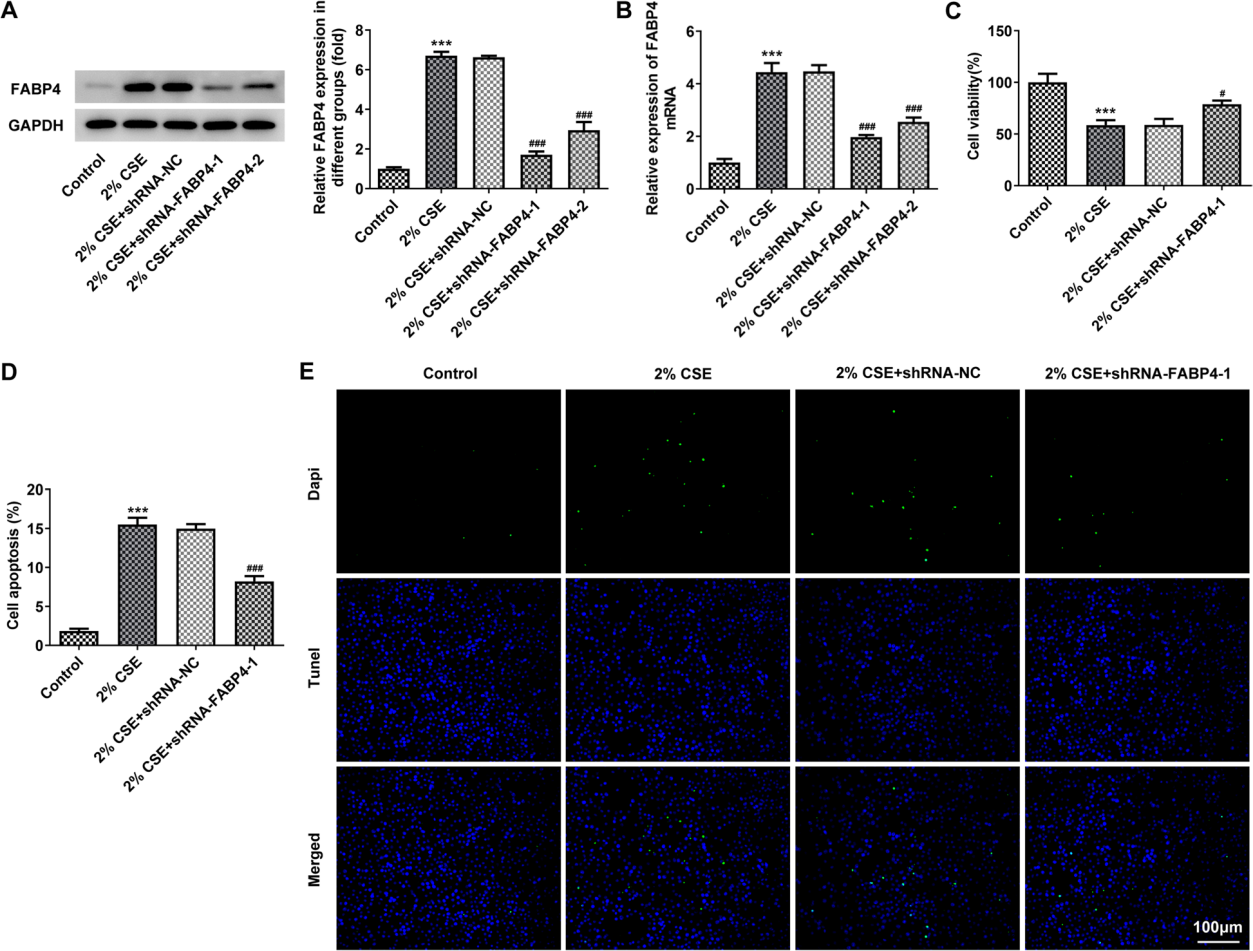
CS-induced 16HBE cell apoptosis is alleviated by shRNA-mediated FABP4 abrogation. **A, B** The knockdown efficiency in 16HBE cells treated with 2% CSE was tested by the western blot analysis and qRT-PCR analysis. GAPDH served as the loading control. **C** CCK-8 assay showed a rescue of CSE-mediated cell viability decrease in 16HBE cells transfected with shRNA-FABP4-1 as compared with the cells transfected with shRNA-NC. **D** TUNEL assay showed a downregulation of CSE-mediated cell apoptosis (TUNEL positive cells/DAPI positive cells; TUNEL, green; DAPI, blue; Scale bar, 100 µm) in 16HBE cells transfected with shRNA-FABP4-1 as compared with the cells transfected with shRNA-NC. The normal 16HBE cells without transfection and CSE treatment were used as control. ***p < 0.001 vs. control group; ^#^*p* < 0.05, ^###^*p* < 0.001 vs. 2% CSE + shRNA-NC group. 16HBE, Human bronchial epithelioid cell line; FABP4, Fatty Acid Binding Protein 4; GAPDH, Glyceraldehyde-3-Phosphate Dehydrogenase; CSE, cigarette smoke extract

To investigate the role of FABP4 in 16HBE cell apoptosis and inflammatory response, shRNAs were synthesized for specific shRNA-mediated FABP4 inhibition after CSE treatment. Western blot and qRT‐PCR analysis showed a remarkable decrease in the FABP4 expression in 2% CSE treated-16HBE cells after transfection of shRNA-FABP4-1/2, comparing with that in 16HBE cells transfected with shRNA-NC (Fig. 2A, B). Also, we observed that shRNA-FABP4-1 exhibited a more effective knockdown efficiency, thus shRNA-FABP4-1 was chosen for the subsequent experiments.

We firstly performed a CCK-8 assay to study the role of FABP4 in CSE-treated 16HBE cells. The result indicated that 16HBE cells exposed to 2% CSE for 48 h showed only 50% of the viability of normal cells while FABP4 abrogation showed an upregulation of viability of 16HBE cells, as compared with that in cells transfected with shRNA-NC (Fig. 2C).

Subsequently, the TUNEL assay was performed to assess the function of FABP4 in CSE-mediated 16HBE cell apoptosis. Notably, after treated with 2% CSE for 48 h, the apoptosis rate (TUNEL positive cells/DAPI positive cells; TUNEL, green; DAPI, blue) was nearly 15%. After transfection of FABP4 specific shRNA, CSE-mediated cell apoptosis was significantly alleviated, and the apoptosis rate decreased to about 8% relative to that in 16HBE cells transfected with shRNA-NC (Fig. 2D).

In conclusion, these results indicate that CSE-mediated FABP4 upregulation reduces the viability and promotes the apoptosis of 16HBE cells.

### FABP4 abrogation alleviates CSE-mediated 16HBE cell inflammatory response and oxidative stress

**Fig. 3:**
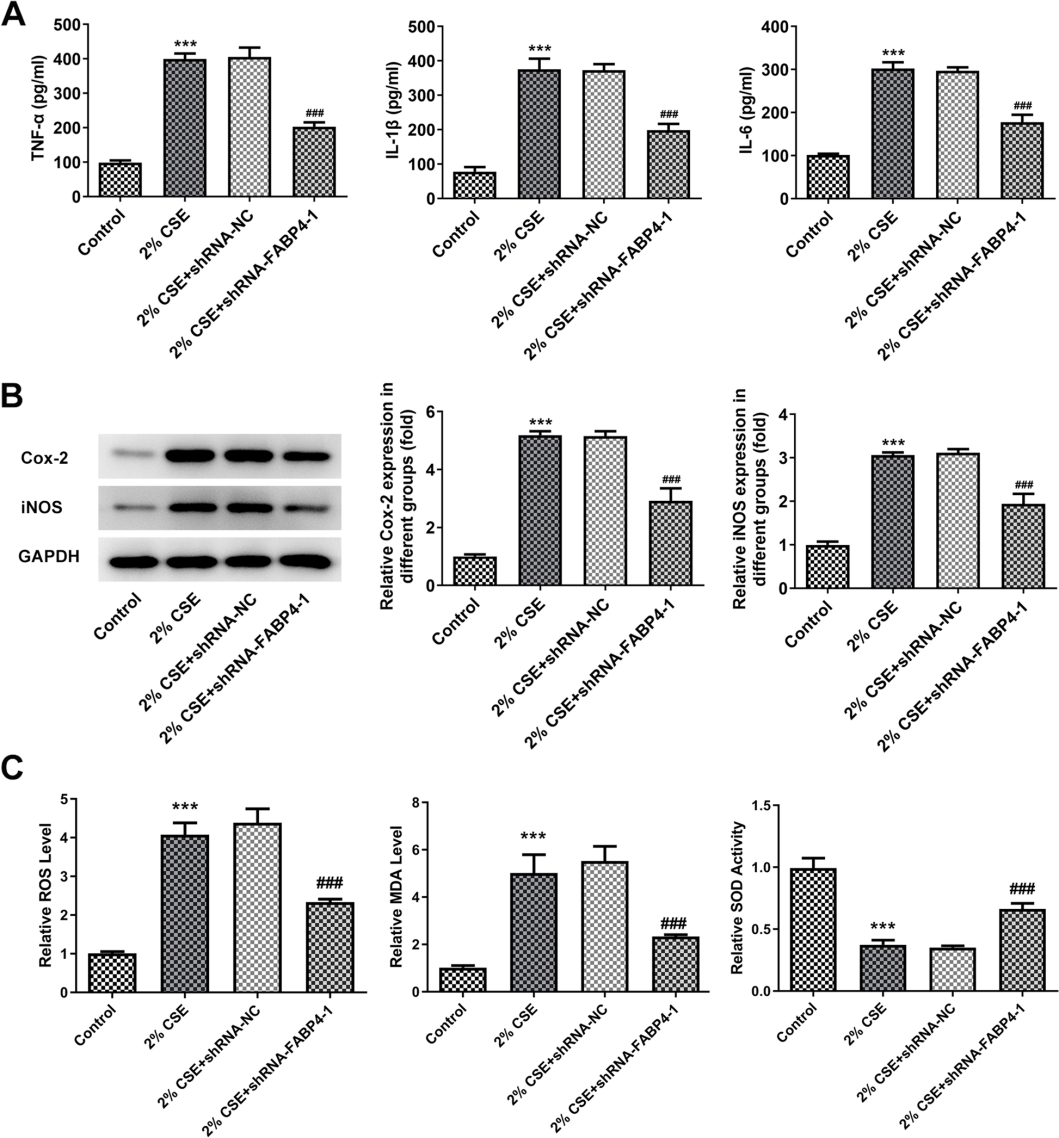
CS-induced 16HBE cell inflammatory response was reduced by shRNA-mediated FABP4 elimination. **A** ELISA assay showed an alleviation of CSE-mediated promotion of TNF-α, IL-1β and IL-6 levels in 16HBE cells transfected with shRNA-FABP4-1 as compared with the cells transfected with shRNA-NC. **B** Western blot analysis showed a downregulation of CSE-mediated increase of Cox-2 and iNOS protein levels in 16HBE cells transfected with shRNA-FABP4-1 as compared with the cells transfected with shRNA-NC. The normal 16HBE cells without transfection and CSE treatment were used as control. ***p < 0.001 vs. control group; ^###^*p* < 0.001 vs. 2% CSE + shRNA-NC group. 16HBE, Human bronchial epithelioid cell line; FABP4, Fatty Acid Binding Protein 4; GAPDH, Glyceraldehyde-3-Phosphate Dehydrogenase; CSE, cigarette smoke extract; TNF-α, Tumor Necrosis Factor α; IL-1β, Interleukin 1 Beta; IL-6, Interleukin 6; Cox-2, Prostaglandin-Endoperoxide Synthase 2; iNOS, Nitric Oxide Synthase 2

After contacting with harmful substances, bronchial epithelial cells are prone to be involved in inflammatory reactions which is caused by cell apoptosis [20, 21]. Hence, we next analyzed levels of inflammatory factors using ELISA assay. Indeed, exposure to 2% CSE dramatically increased levels of the cellular inflammatory factors including TNF-α, IL-1β and IL-6. In 16HBE cells transfected with FABP4 specific shRNA, although the same 2% CSE treatment was accomplished for 48 h, the levels of inflammatory factors were much lower than that in cells transfected with shRNA-negative control (NC) (Fig. 3A). Cyclooxygenase-2 (Cox-2) and inducible nitric oxide synthase (iNOS) are closely linked and are identified as pro-inflammatory cytokines. Therefore, we used western blot analysis to test the protein levels of Cox-2 and iNOS and the results confirmed that FABP4 inhibition reduced CSE-mediated cellular inflammatory response in 16HBE cells (Fig. 3B). In addition, the changes in oxidative stress were assessed. As shown in Fig. 3C, exposure to 2% CSE caused a significant increase in ROS and MDA level but decrease in SOD activity, suggesting the induction of oxidative stress. However, upon FABP4 silence, ROS and MDA levels were reduced and SOD activity was enhanced (Fig. 3C).

Taken together, these findings suggest that FABP4 knockdown in 16HBE cells alleviates CSE-mediated inflammatory response and oxidative stress.

### FABP4 silence inhibits 16HBE cell inflammatory response, oxidative stress and apoptosis by inactivating p38 MAPK/MK2 signaling pathway

**Fig. 4:**
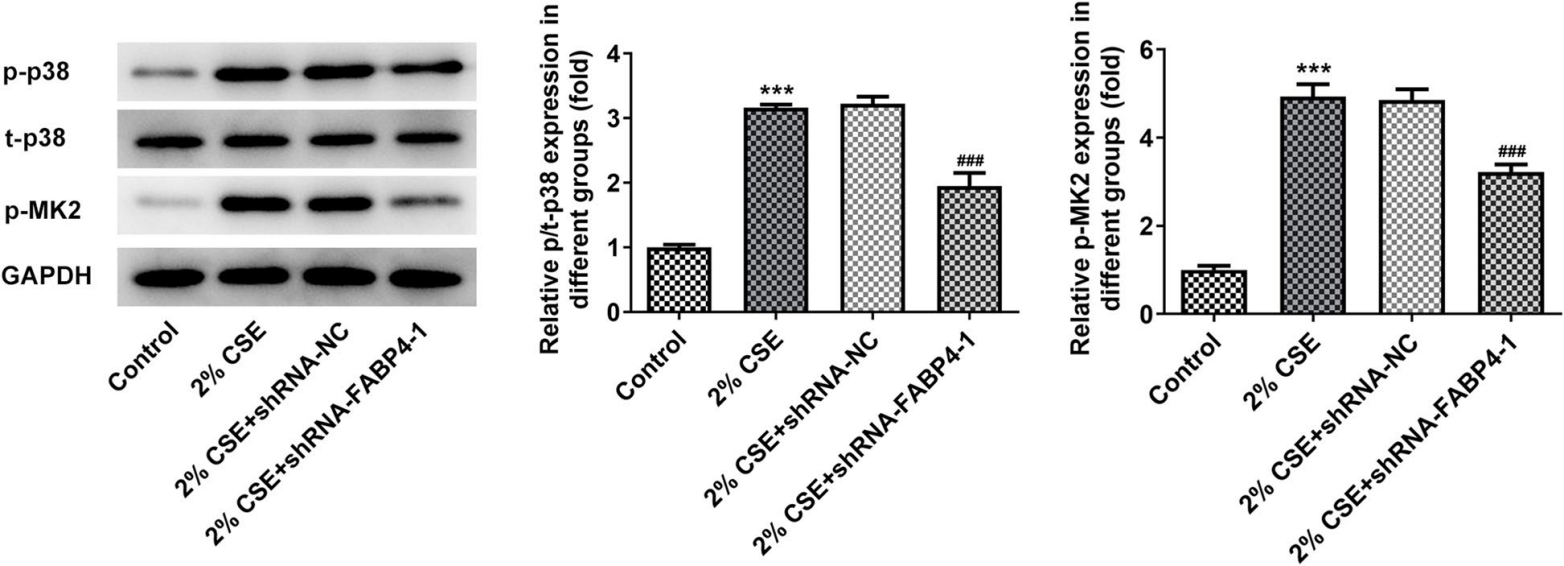
Interference of FABP4 inhibits p38 MAPK/MK2 signaling pathway in 16HBE cells. Western blot analysis showed a suppression of CSE-mediated activation of p38 MAPK/MK2 signaling pathway in 16HBE cells transfected with shRNA-FABP4-1 as compared with the cells transfected with shRNA-NC. The normal 16HBE cells without transfection and CSE treatment were used as control. ***p < 0.001 vs. control group; ^###^*p* < 0.001 vs. 2% CSE + shRNA-NC group. 16HBE, Human bronchial epithelioid cell line; FABP4, Fatty Acid Binding Protein 4; p38, Mitogen-Activated Protein Kinase 14; p-p38, (Thr180/Tyr182); MK2, MAPK Activated Protein Kinase 2; p-MK2, (Thr334); GAPDH, Glyceraldehyde-3-Phosphate Dehydrogenase; CSE, cigarette smoke extract

As reported, MAPK is activated in human bronchial epithelial cells exposed to CSE [22]. To explore whether FABP4 was involved in the p38 MAPK/MK2 signaling pathway in CSE-mediated 16HBE cell inflammatory response and apoptosis, we used western blot analysis to detect related proteins in this pathway. As predicted, phosphorylation levels of P38 and MK2 were significantly increased in 16HBE cells after 2% CSE treatment for 48 h. In FABP4-deficient cells, phosphorylation levels of P38 and MK2 were limited approximately 40% (Fig. 4).

**Fig. 5:**
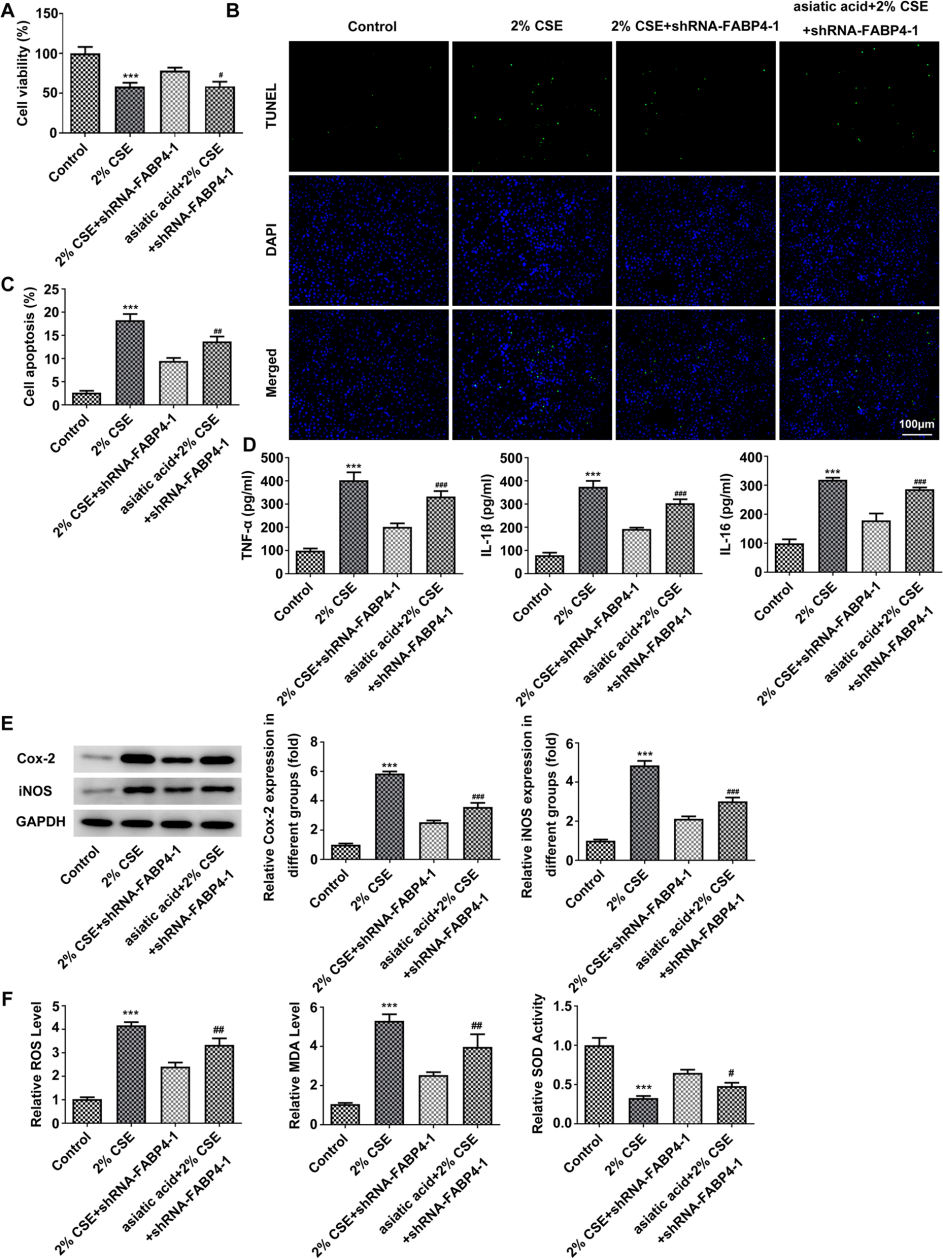
CS-induced 16HBE cell inflammatory response and apoptosis are generated by FABP4-mediated activation of p38 MAPK/MK2 signaling pathway. **A** CCK-8 assay showed that shRNA-mediated FABP4 ablation rescued the CSE-mediated cell viability decline, (**B-C**) TUNEL assay showed that shRNA-mediated FABP4 inhibition downregulated the CSE-mediated cell apoptosis (TUNEL positive cells/DAPI positive cells; TUNEL, green; DAPI, blue; Scale bar, 100 µm), (**D**) ELISA assay showed that shRNA-mediated FABP4 abrogation alleviated the CSE-mediated cellular inflammatory response (TNF-α, IL-1β and IL-6), (**E**) Western blot analysis showed that shRNA-mediated FABP4 abolition downregulated the CSE-mediated Cox-2 and iNOS protein levels, which were all significantly reversed after treatment with 30 μM asiatic acid, an agonist of p38 MAPK at 37 °C for 1 h. The normal 16HBE cells without transfection and CSE or asiatic acid treatment were used as control. ***p < 0.001 vs. control group; ^#^*p* < 0.05, ^##^*p* < 0.01, ^###^*p* < 0.001 vs. 2% CSE + shRNA-FABP4-1 group. 16HBE, Human bronchial epithelioid cell line; FABP4, Fatty Acid Binding Protein 4; GAPDH, Glyceraldehyde-3-Phosphate Dehydrogenase; CSE, cigarette smoke extract; TNF-α, Tumor Necrosis Factor α; IL-1β, Interleukin 1 Beta; IL-6, Interleukin 6; Cox-2, Prostaglandin-Endoperoxide Synthase 2; iNOS, Nitric Oxide Synthase 2

To further confirm the FABP4 signaling pathway involved in CSE-mediated 16HBE cell inflammatory response and apoptosis, asiatic acid, an agonist of p38 MAPK was used [23]. After pretreated with 30 μM asiatic acid for 1 h, the protective effects of FABP4 ablation against CSE exposure-mediated 16HBE cell injury were inhibited, with cell viability was decreased to about 50% (Fig. 5A) and cell apoptosis was increased to about 14% relative to those of the 2% CSE + shRNA-FABP4-1 group (Fig. 5B, C). At this time, through ELISA assay and western blot analysis, the levels of inflammatory factors including TNF-α, IL-1β and IL-6 were slightly increased (Fig. 5D), and the levels of inflammatory proteins Cox-2 and iNOS were also increased by asiatic acid (Fig. 5E), as compared with those in 2% CSE + shRNA-FABP4-1 treated 16HBE cells. In addition, upon asiatic acid treatment, ROS and MDA levels were increased and SOD activity was decreased when compared with 2% CSE + shRNA-FABP4-1 treatment (Fig. 5F).

Thus, we conclude that upregulation of FABP4 promotes CSE-mediated 16HBE cell injury by activating the P38 MAPK/MK2 signaling pathway.

## Discussion

As early as 1997, a research report has pointed out that exposure to CS induces airway inflammation in smokers by inducing the release of IL-8 from bronchial epithelial cells [24]. Direct exposure to CSE significantly reduces the production of vascular endothelial growth factor in well-differentiated primary human airway epithelial cells by changing the extracellular signal-regulated kinase 1/2 and protein kinase C signaling pathways [25]. Here, in this study, we aim to explore the potential link between CSE exposure and FABP4 expression in bronchial epithelial cells. Interestingly, our in vitro results showed that FABP4 expression was increased in a time- and dose-dependent manner in CSE-treated 16HBE cells, a human bronchial epithelial cell line. FABP4 mainly plays a role in regulating metabolism and inflammation pathways [17, 26–28]. Chlamydia pneumoniae in mice infects and proliferates fat cells by inducing hormone-sensitive lipase-mediated lipolysis [7], and strongly induces fat cells to secrete FABP4 by stimulating endoplasmic reticulum stress and unfolded protein response [6]. In addition, increased levels of FABP4 have also been found in inflammation-induced bronchopulmonary dysplasia and hyperoxia-induced lung injury [8, 9]. These literatures and research results imply the potential relationship among CS, FABP4 up-regulation and bronchial epithelial cell inflammation.

It is commonly reported that CSE causes chronic lung disease by changing the spectrum of inflammatory cells [29, 30]. We next carried out ELISA assay and western blot analysis to detect the levels of the cellular inflammatory factors including TNF-α, IL-1β and IL-6 and the protein levels of pro-inflammatory cytokines including Cox-2 and iNOS. Elevated Cox-2 and iNOS expressions has been confirmed to be associated with the development of chronic lung diseases leading including COPD [31, 32]. Consistent with these findings, the results showed that CSE exposure elevated the levels of TNF-α, IL-1β and IL-6 and the protein levels of Cox-2 and iNOS in 16HBE cells. Also, the ablation of FABP4 in 16HBE cells alleviated the CSE-mediated inflammatory response.

Moreover, CSE is known to induce oxidative stress in COPD models both in vitro and in vivo [33, 34]. Consistently, by observing the level of oxidative stress markers including ROS, MDA and SOD, we found that CSE remarkably promoted oxidative stress in 16HBE cells. Besides, FAPB4 inhibitor was reported to attenuate oxidative stress in human alveolar epithelial A549 cells, suggesting the promotive effect of FABP4 on oxidative stress in respiratory endothelium [10]. We subsequently detected the influence of FABP4 on the oxidative stress in CSE-treated 16HBE cells, and found that CSE-mediated increase in oxidative stress in 16HBE cells was markedly blocked by FABP4 knockdown. This data suggested the inhibitory effect of FABP4 knockdown on CSE-mediated oxidative stress.

In the process of atherosclerosis, Gao Q et al. have found that continuously activated FABP4 mediates endoplasmic reticulum stress and macrophage apoptosis [35]. Moreover, the up-regulation of FABP4 can cause spontaneous apoptosis in bone marrow adipocytes [36]. However, Elmasri H et al. have found that the aortic ring of FABP4-deficient mice shows reduced angiogenesis, and FABP4 deficiency are prone to stimulate the apoptosis of human umbilical vein endothelial cells [26]. Therefore, we conclude that the regulation of cell survival by FABP4 may differ in different cell types. In this study, we discovered that shRNA-mediated FABP4 specific inhibition rescued the decrease in 16HBE cell activity and the increase in apoptosis caused by CSE exposure.

It is previously mentioned that FABP4 upregulation induces the apoptosis of bone marrow adipocytes by activating p38 MAPK signaling pathway [26]. In adipocytes, exogenous FABP4 can interfere with adipogenic differentiation through p38 MAPK-mediated lipolysis and inflammation both in vivo and in vitro [27]. In addition, MK2, a known downstream substrate of MAPK, is phosphorylated in CSE-induced bronchial epithelial cells [15]. Another study also clarified that p38 inhibition attenuates the expression of IL-6, iNOS, and Cox-2 [36]. Our experimental results confirmed that silencing of FABP4 can inhibit the activation of p38 MAPK/MK2 signaling pathway in 16HBE cells. More importantly, asiatic acid, an agonist of p38 MAPK, reversed the protective role of FABP4 reduction against 16HBE cell injury upon exposure to CSE. These results indicated that CSE-mediated up-regulation of FABP4 expression exerted pro-inflammatory, pro-oxidative stress and pro-apoptotic effects by activating the p38 MAPK/MK2 signaling pathway in 16HBE cells. However, the usage of only one cell line and the lack of in vivo experiments are limitations of this study, we may consider to validate our findings in other cell line models and animal models in research work. In addition, FABP4 has been reported to induce asthmatic airway epithelial barrier dysfunction via regulating ROS [37], while this study didn’t cover this issue. Furthermore, asiatic acid did not reverse the effect of FABP4 silence totally, indicating the possible involvement of other pathways in the actions of FABP4, which remains to be elucidated. Therefore, our future study aims to give an explanation of FABP4 role in bronchial epithelial cells in a more comprehensive perspective.

## Conclusion

In a word, our results highlight a novel finding that the expression of FABP4 was increased in a time and dose-dependent manner in 16HBE cells exposed to CSE. The knockdown of FABP4 protect bronchial epithelial cells against CSE-induced injury via inactivating p38 MAPK/MK2 signaling pathway. Our results provide a novel markers or target for the development of therapies for COPD.

## Materials and methods

### Preparation of CSE

100% CSE was prepared by allowing the smoke generated by two 3R4F standard research cigarettes (Tobacco and Health Research Institute, University of Kentucky) with filter removed to flow directly into RPMI-1640 (sigma) in a glass flask with side flow smoke exposure system. Each cigarette burns for 8 min. The absorbance value of the collected 100% CSE was measured at the wavelength of 260 nm for inter batch quality control. The qualified 100% CSE was filtered and sterilized with 0.22 μm filter for the experiment.

### Cell culture

Studies were performed using human bronchial epithelioid cell line 16HBE cells purchased from American type culture collection (ATCC). Cells were maintained in RPMI-1640 containing 10% Fetal Bovine Serum (FBS; Gibco), 0.1kU/ mL penicillin and 0.1 mg/ml streptomycin (Beyotime Biotechnology) at 37 °C in a humidified 5% CO2 atmosphere. The passage number of all cells used has no more than 10.

### Cell transfection

FABP4-specific short hairpin RNAs (shRNA; shRNA-FABP4-1, shRNA-FABP4-2) and nontargeting shRNA (shRNA-NC) were synthesized and purified by RiboBio. Briefly, 5 × 10^5^ 16HBE cells were transfected with 25 pmol shRNA using Lipofectamine® RNAiMAX (Invitrogen) according to manufacturer’s recommendation.

Sequences are as follows: shRNA-FABP4-1, sense 5’- GGATGTGATCACCATTAAA-3’, antisense 5’- TTTAATGGTGATCACATCC-3’; shRNA-FABP4-2, sense 5’- GGGATGTGATCACCATTAA-3’, antisense 5’- TTAATGGTGATCACATCCC-3’; shRNA-NC, sense 5’-GATCCCCCTTCTCCGAACG-3’, antisense 5’-AGCTAAAAATTCTCCGAAC-3’.

### RNA extraction, complementary DNA (cDNA) synthesis, real‐time quantitative reverse transcription‐polymerase chain reaction (qRT‐PCR)

Total cellular RNA was isolated from 5 × 10^6^ 16HBE cells using the Total RNA Isolation Kit (Biomarker), and 5 µg of total RNA was used to reverse into cDNA using the PrimeScript™ RT reagent kit (Takara), according to manufacturers’ recommendations. Subsequently, the qRT‐PCR were performed using One Step TB Green® PrimeScript™ PLUS RT-PCR Kit (Takara) and detected by StepOnePlus™ Real-Time PCR System (Thermo Fisher Scientific).

Primer sequences were as follows: for human FABP4, forward 5’-ATGGGGGTGTCCTGGTACAT-3’, reverse 5’-CTTTCATGACGCATTCCACCA-3’; for human GAPDH, forward 5’-CACTAGGCGCTCACTGTTCT-3’, reverse 5’-GCCCAATACGACCAAATCCGT-3’.

### Western blot (WB) analysis

Total cellular protein was prepared from 1 × 10^7^ 16HBE cells using 1 ml CelLytic™ MT Cell Lysis Reagent (Sigma) containing Phosphatase Inhibitor Cocktail (abcam). The proteins (20 µg/lane) were separated using 10% sodium dodecyl sulfate–polyacrylamide gel electrophoresis (SDS-PAGE) and transferred onto 0.45 µm polyvinylidene fluoride (PVDF) membranes (Millipore), and blocked with Tris-buffered saline containing 1% Tween-20 (TBST, pH 7.6) and 5% skimmed milk powder at room temperature for 1 h. After washed with TBST, the blots were incubated with primary antibodies at 4 °C overnight. After additional three more washes with PBS (15 min each time), the corresponding horseradish peroxidase (HRP)-linked secondary antibodies were used at room temperature for another 1 h. Finally, the protein bands were visualized with Chemiluminescent Western Blot Reagents (Thermo Fisher Scientific) and scanned with the PDQuest 7.2.0 software (Bio-Rad). Protein expression was quantified using ImageJ software (version 1.8.0; National Institutes of Health).

Primary antibodies: rabbit anti-FABP4 (1:1,000, 2120S, Cell Signaling technology); rabbit anti-GAPDH (1:1,000, 5174S, Cell Signaling technology); rabbit anti-Cox-2 (1:1,000, 12282S, Cell Signaling technology); rabbit anti-iNOS (1:1,000, 13120S, Cell Signaling technology); rabbit anti-p38 (1:1,000, 8690S, Cell Signaling technology); rabbit anti-p-p38 (1:1,000, 4511S, Cell Signaling technology); rabbit anti-p-MK2 (1:1,000, 3041S, Cell Signaling technology).

HRP-linked secondary antibody: Anti-rabbit IgG, HRP-linked Antibody (1:3,000, 7074S, Cell Signaling technology).

### Cell Counting Kit-8 (CCK-8) assay

1 × 10^4^ 16HBE cells/well were seeded into 96-well plates and transfected with corresponding shRNA at 37 °C for 24 h. After 1 h treatment with or without 30 μM asiatic acid, an agonist of p38 MAPK at 37 °C, 100 µl fresh RPMI-1640 completement medium containing 2% CSE were replaced and cultured for additional 48 h. Subsequently, Cell Counting Kit-8 (MedChem Express) was used to determine the cell viability according to the manufacturer’s recommendation. The normal 16HBE cells without transfection and treatment were used as control.

### Tunel assay

5 × 10^4^ 16HBE cells/well were seeded into 96-well plates and transfected with corresponding shRNA at 37 °C for 24 h. After 1 h treatment with or without 30 μM p38 agonist asiatic acid at 37 °C, 500 µl fresh RPMI-1640 completement medium containing 2% CSE were replaced and cultured for additional 48 h. Subsequently, One Step TUNEL Apoptosis Assay Kit (Beyotime Biotechnology) was used to determine the cell apoptosis according to the manufacturer’s recommendation. The normal 16HBE cells without transfection and treatment were used as control. The percentage of apoptotic cells was calculated using ImageJ software (version 1.8.0; National Institutes of Health).

### Elisa assay

2 × 10^5^ 16HBE cells were seeded into cell culture dishes and transfected with corresponding shRNA at 37 °C for 24 h. After 1 h treatment with or without 30 μM asiatic acid, an agonist of p38 MAPK at 37 °C, 8 ml fresh RPMI-1640 completement medium containing 2% CSE were replaced and cultured for additional 48 h. Subsequently, Human TNF alpha SimpleStep ELISA® Kit, Human IL-1 beta SimpleStep ELISA® Kit, Human IL-6 SimpleStep ELISA® Kit (abcam) were used to determine the levels of inflammatory cytokines in culture supernatant according to the manufacturer’s commendation. The normal 16HBE cells without transfection and treatment were used as control.

### Measurement of oxidative stress

Production of ROS and MDA, and SOD activity in 16HBE cells was measured by Cellular ROS Detection Assay Kit (ab186029; Abcam), Lipid Peroxidation (MDA) Assay Kit (ab118970; Abcam) and SOD Activity Assay Kit (ab65354; Abcam), respectively, strictly according to the manufactures’ protocols. Briefly, control or transfected 16HBE cells were treated with or without 30 μM asiatic acid for 1 h, followed by being cultured in fresh RPMI-1640 medium containing 2% CSE for additional 48 h. Subsequently, about 2 × 10^6^ cells were harvested for corresponding Assay Kits. All experiments were performed in triplicate and data were shown as relative level or activity after normalization to control group.

### Statistics

All data were shown as the mean ± standard deviation from experiments in triplicate. Comparisons between multiple groups were evaluated using one-way ANOVA followed by Tukey’s test using GraphPad Prism 8. *P* value < 0.05 was considered significant.

## Data Availability

The datasets used and/or analyzed during the current study are available from the corresponding author on reasonable request.

## Abbreviations

CSE: Cigarette Smoke Extract
FABP4: Fatty Acid Binding Protein 4
ROS: Reactive Oxygen Species
COPD: Chronic Obstructive Pulmonary Disease
IL-6: Interleukin 6
MAPK: Mitogen-Activated Protein Kinase
MK2: MAPK activated kinase 2
ATCC: American Type Culture Collection
shRNA-FABP4: FABP4-specific short hairpin RNA
shRNA-NC: Nontargeting shRNA
cDNA: Complementary DNA
qRT‐PCR: Real‐Time Quantitative Reverse Transcription‐Polymerase Chain Reaction
WB: Western Blot
CCK-8: Cell Counting Kit-8,
16HBE: Human Bronchial Epithelioid Cell Line
GAPDH: Glyceraldehyde-3-Phosphate Dehydrogenase
TNF-α: Tumor Necrosis Factor α
IL-1β: Interleukin 1 Beta
Cox-2: Prostaglandin-Endoperoxide Synthase 2
iNOS: Nitric Oxide Synthase 2
p38: Mitogen-Activated Protein Kinase 14
p-p38: (Thr180/Tyr182)
MK2: MAPK activated protein kinase 2
p-MK2: (Thr334)
Cox-2: Prostaglandin-Endoperoxide Synthase 2
iNOS: Nitric Oxide Synthase 2
